# 

**Published:** 1856-11

**Authors:** 


### RECEIPTS ON SUBSCRIPTIONS,

UP TO OCT. 30TH, 1856.

### ILLINOIS.

| | | | |
|---|---|---|---|
| Dr. | J. C. Brown, | Vol. 4&5, | $4,00 |
| " | J. F. Spellman, | " 5, | 2,00 |
| " | G. W. Albin, | " 5, | 2,00 |
| " | P. A. Rosenberger, | " 5, | 2.00 |
| " | Max Myers, | " 4&5, | 4,00 |
| " | C. H. Quinlan, | " 4&5, | 4,00 |
| " | J. C. Morfit, | " 4&5, | 4,00 |
| " | Henrotin, | " 4&5, | 4,00 |
| " | W. W. Snowden, | " 4&5, | 4,00 |
| " | N. Holton, | " 5, | 2,00 |
| " | Benj. Woodward, | " 5, | 2,00 |
| " | J. Emery, | " 6, | 2,00 |
| " | J. R. Snelling, | " 5, | 2,00 |
| " | J. B. Samuel, | " 5, | 2,00 |

### WISCONSIN.

| | | | |
|---|---|---|---|
| Dr. | E. G. Dyer, | Vol. 5, | $2,00 |
| " | S. S. Clark, | " 5, | 2,00 |
| " | Hays McKinlay, | " 5, | 2,00 |

### MICHIGAN.

| | | | |
|---|---|---|---|
| Dr. | J. S. Skinner, | Vol. 5, | $2,00 |

### IOWA.

| | | | |
|---|---|---|---|
| Dr. | A. Boomer, | Vol. 5, | $2,00 |
| " | J. Doron, | " 5, | 2,00 |
| " | E. A. Smith, | " 5, | 2,00 |

### INDIANA.

| | | | |
|---|---|---|---|
| Dr. | M. R. Chadwick, | Vol. 5, | $2,00 |
| " | J. B. Buchlett, | " 4&5, | 4,00 |
| " | U. A. V. Hester, | " 4&5, | 4,00 |

### OHIO.

| | | | |
|---|---|---|---|
| Dr. | J. B. Evans, | Vol. 4&5, | $4,00 |

### MINNESOTA TERRITORY.

| | | | |
|---|---|---|---|
| Dr. | J. R. Dart, | Vol. 6, | $2,00 |

